# Synthesis and Characterization of Polycarbonates by Melt Phase Interchange Reactions of Alkylene and Arylene Diacetates with Alkylene and Arylene Diphenyl Dicarbonates

**DOI:** 10.3390/molecules15053661

**Published:** 2010-05-18

**Authors:** Bassam A. Sweileh, Yusuf M. Al-Hiari, Mohammad H. Kailani, Hani A. Mohammad

**Affiliations:** 1 Department of Chemistry, Faculty of Science, The University of Jordan, Amman 11942, Jordan; E-Mail: kailani@ju.edu.jo (M.H.K.); 2 Department of Pharmaceutical Sciences, Faculty of Pharmacy, The University of Jordan, Amman 11942, Jordan; E-Mail: hiary@ju.edu.jo (Y.M.A.-H.); 3 Department of Chemistry, Faculty of Arts and Sciences, University of Petra, P.O. Box 961343, Amman 11196, Jordan; E-Mail: hanimohammad@uop.edu.jo (H.A.M.)

**Keywords:** diphenyldicarbonate, diacetates, polycondensation, polycarbonates, synthesis, interchange reactions

## Abstract

This work presents a new synthetic approach to aromatic and aliphatic polycarbonates by melt polycondensation of bisphenol A diacetates with alkylene- and arylenediphenyl dicarbonates. The diphenyl dicarbonates were prepared from phenyl chloroformate and the corresponding dihydroxy compounds. The process involved a precondensation step under a slow stream of dry argon with the elimination of phenyl acetate, followed by melt polycondensation at high temperature and under vacuum. The potential of this reaction is demonstrated by the successful synthesis of a series of aromatic-aromatic and aromatic-aliphatic polycarbonates having inherent viscosities from 0.19 to 0.43 dL/g. Thus low to intermediate molecular mass polymers were obtained. The ^13^C-NMR spectra of the carbon of the carbonate group showed that the formed polycarbonates contain partial random sequence distribution of monomer residues in their chains. The polycarbonates were characterized by inherent viscosity, FTIR, ^1^H-NMR and ^13^C-NMR spectroscopy. The glass transition temperatures, measured by DSC, of the polycarbonates were in the range 13–108 °C. The thermogravimetric curves of showed that these polymers have good thermal stability up to 250 °C. The present approach may open the door for novel polycarbonates containing other organic functional groups.

## 1. Introduction

Polycarbonates are high heat engineered thermoplastic polymers having unique properties. They are characterized by outstanding mechanical, optical and thermal properties and have a wide range of applications [1,2,3,4,5,6,7,8]. Aromatic polycarbonates derived from bisphenol A have been extensively studied and found to be interesting because of their useful properties; such as rigid molecular structure, exceptional impact resistance, chemical and dimensional stability, toughness, optical clarity, and thermal stability [6,7,8,9,10,11,8,9]. They also offer excellent moldability and extrudability, good fire resistance, dimensional stability, and high optical transparency opening the door for a wide range of industrial applications [1,12]. Other properties such as modulus, dielectric strength and tensile strength are comparable to other amorphous thermoplastics at similar temperatures below their respective glass transition temperatures (T_g_s). However, while most amorphous polymers are stiff and brittle below their T_g_ values, polycarbonates retain their ductility and impact resistance below their T_g_ values [1].

Polycarbonates are prepared industrially by two different processes: 1) Schotten-Baumann reaction of phosgene with an aromatic dihydroxy compound in an amine-catalyzed interfacial polycondensation reaction and 2), base-catalyzed transesterification of a bisphenol with diphenyl carbonate [1,3,4,13]. The first process uses phosgene, which is known for its high toxicity and lethality, whereas dichloromethane is suspected to be a carcinogen and a source of chlorine in producing dioxin [14]. The method also produces sodium chloride wastes contaminated with organic substances [15]. Since there is an increasing pressure to avoid using chlorinated organic solvents in the chemical industry, the search for environmentally benign synthetic routes to polycarbonates that don’t involve phosgene and chlorinated solvents is still required [2,4,14,16]. The second process utilizes diphenyl carbonate (DPC), now prepared by a variety of nonphosgene methods [1,17,18] in a melt phase polycondensation reaction. The high temperatures of the reaction melt, the low pressures, the high viscosity of the polymer melt, the difficulty to regulate the molecular weight and the side reactions that give a slightly yellowish color to the product are major drawbacks of this process [1,3,4].

Numerous polycarbonates based on bisphenol A (BPA) and other diphenols and aliphatic diols have been prepared to improve the physical properties of homopolycarbonates. Among the main variations carried on polycarbonates was the synthesis of liquid crystalline polycarbonate as poly(alkylene carbonate)s. Aliphatic polycarbonates have attracted lesser attention as structural materials compared to aromatic polycarbonates due to their poor mechanical properties. Nevertheless, they show potential as biodegradable and biocompatible materials for drug delivery systems [5,19,20,21]. Aromatic–aliphatic polycarbonates are a combination of aliphatic and aromatic components to improve the flexibility and elasticity of aromatic polycarbonates and enhancing the poor physical properties of aliphatic polycarbonates [22].

Alkylene- and arylenediphenyl dicarbonates were used as monomers reacting with aliphatic and aromatic dihydroxy compounds by melt polycondensation for the synthesis of polycarbonates. Several studies used this reaction for the synthesis and investigation of thermotropic liquid crystalline properties of homo-, co-, and terpolycarbonates prepared by this method [23,24,25,26,27,28,29,30,31,32]. Liaw and coworkers [9,33,34] used selected arylene- and alkylenediphenyl dicarbonats (e.g., bisphenol AF diphenyl dicarbonate) to prepare copolycarbonates via melt polycondensation with bisphenols (e.g., bisphenol S) and studied their thermal properties.

Many literature methods have been reported for the preparation of polycarbonates utilizing bisphenol A diaceatate as a starting monomer. The methods comprise reactions with dicarboxylic acid dimethyl esters and diol dicarbonates [35] dialkyl carbonates in the melt [2] or with dialkyl or diaryl carbonate in presence of an inert high boiling solvent [36,37,38,39].

We have recently been interested in the melt-phase interchange process for the preparation of polycarbonates from alkylene- and arylenediphenyl dicarbonates and dihydroxy compounds in an effort to direct the technology to environmentally friendly processes [22,40]. In this context, the reported literature utilizing BPA diacetate (BPAAc2) involves reaction with dimethyl carbonate (DMC) [2]. In the course of our program of work involving the study of preparation of polycarbonate, we investigated the synthesis of polycarbonates by a different reaction. Thus the synthesis of polycarbonates from alkylene- or arylenediacetate reacting with alkylene- or arylenediphenyl dicarbonates instead of DMC was examined. This approach is completely novel and has no precedent in the literature. Therefore, the objective of this work was to study a new synthetic method to polycarbonates by melt-phase polycondensation of alkylene- and arylenediacetate with alkylene- and arylenediphenyl dicarbonates. The advantage of this reaction is that it permits varying the structures of the alkylene and arylene units in both monomers which would lead to polymers with widely varying structures and properties (e.g., thermal properties, *i.e.* T_g_). This article presents the results of this study.

## 2. Results and Discussion

### 2.1. Synthesis and characterization of monomers

**Scheme 1:**
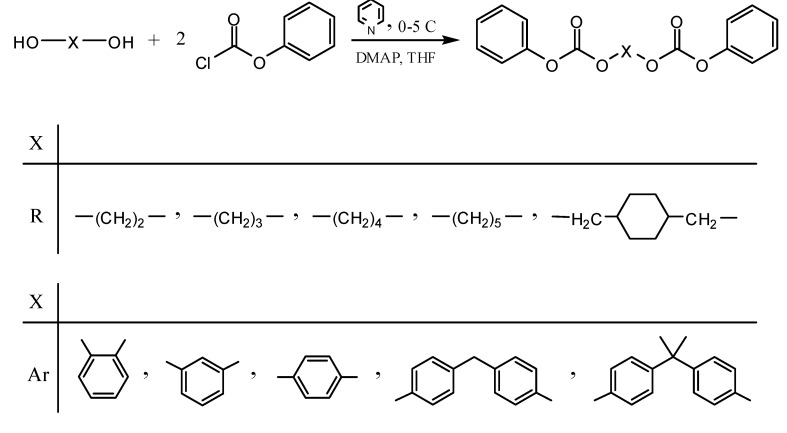
Synthesis of alkylene- and arylenediphenyldicarbonate monomers.

The synthetic approach used in this work was based on interchange reaction of alkylene- or arylene- diacetates with alkylene- or arylenediphenyl dicarbonates. Bisphenol A diacetate (BPAAc2) used was a commercial sample. 1,4-Cyclohexanedimethylene diacetate (CHDMAc2) was prepared from 1,4-cyclohexanedimethanol (CHDM) and acetyl chloride in the presence of excess pyridine in chloroform solution. The alkylene- and arylenediphenyl dicarbonates were prepared in THF solution by the reaction of the various dihydroxy compounds with phenyl chloroformate (PCF) in the presence of excess pyridine [33] as shown in Scheme 1. The structures of 1,4-cyclohexanedimethylene diacetate and the diphenyl dicarbonate monomers, which were obtained in high yields as white solids or slightly viscous liquids, were confirmed by melting point, IR spectroscopy, ^1^H-NMR and ^13^C-NMR spectroscopy. The yields and the physical properties of the diacetate and the alkylene- and arylene- diphenyl dicarbonate monomers are presented in Table 1.

**Table 1:** The yield and physical properties of alkylene- and arylenediphenyldicarbonate and diacetate monomers.

	Yield (%)	T_m_ (°C)	C-O-C (cm^-1^)	C=O (cm^-1^)
**Diphenyl dicarbonates**				
1,2- Ethanediol	92.7	94	1209, 1257	1757
1,3-Propanediol	87.5	52	1210, 1242	1761
1,4-Butanediol	89.8	82	1205, 1260	1754
1,5-Pentanediol	94.4	52	1210, 1252	1760
1,4-Cyclohexanedimethanol	90.2	145–150	1208, 1260	1755
Catechol	83.3	100–103	1197, 1245	1787
Resorcinol	85.1	126	1128, 1163, 1234	1775
Hydroquinone	87.1	124	1271	1769
4,4'-Bis(hydroxyphenyl)methane	91.6	97–100	1158, 1184, 1253	1768
Bisphenol A	84.4	90	1170, 1189, 1235	1775
**Diacetates**				
Bisphenol A diacetate	—	89–91	1170, 1210	1755
1,4-Cyclohexanedimethanol diacetate	87	70	1239	1741

**Figure 1:**
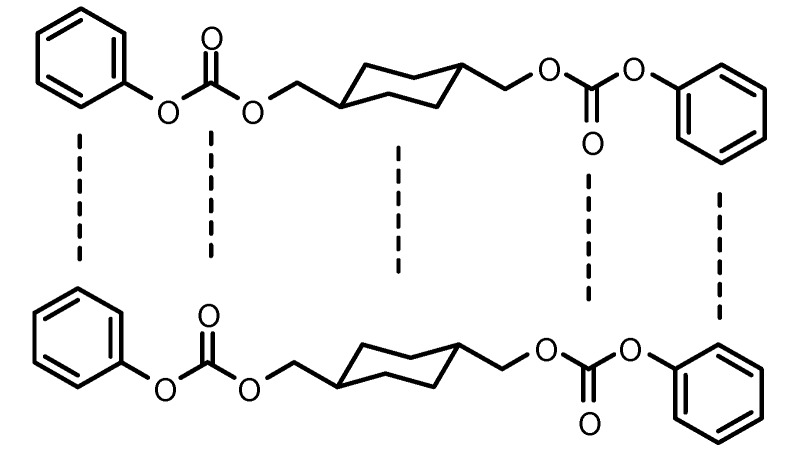
The structure of CHDM DPDC showing the various planar molecular sections.

The diacetate and diphenyl dicarbonate monomers were obtained in high yields. The melting points of the alkylene- and arylenediphenyl dicarbonates were in the range 52–150 °C. 1,3-Propanediol diphenyl dicarbonate (PrD DPDC) and 1,5-pentanediol diphenyl dicarbonate (PeD DPDC), which have odd-carbon numbers in the alkane chain, showed the lowest melting points of all of the synthesized diphenyl dicarbonates. Even-carbon number alkylenes diphenyl dicarbonates fit better in their crystal lattices than odd ones probably due to their structural symmetry and thus exhibited slightly higher melting points. On the other hand, 1,4-cyclohexanedimethylene diphenyl dicarbonate (CHDM DPDC) showed unexpectedly the highest melting point among the diphenyl dicarbonates prepared. This fact may have been due to that the molecule contains a number molecular sections (*i.e.*, the chair cyclohexane ring, the carbonate group and the phenyl group) which can fit very well to the corresponding planes is another molecule, so they can stack easily in their crystal lattice. It seems that the chair conformation of the cyclohexylene ring although not planar it permits good packing. It has been reported that the incorporation of trans-1,4-cyclohexylene units in the backbone of glassy poly(ester carbonate)s improves chain packing due to ring inversion and the shape of the trans 1,4-cyclohexylene rings [41] (Figure 1).

#### 2.1.1. Infrared spectroscopy

**Figure 2:**
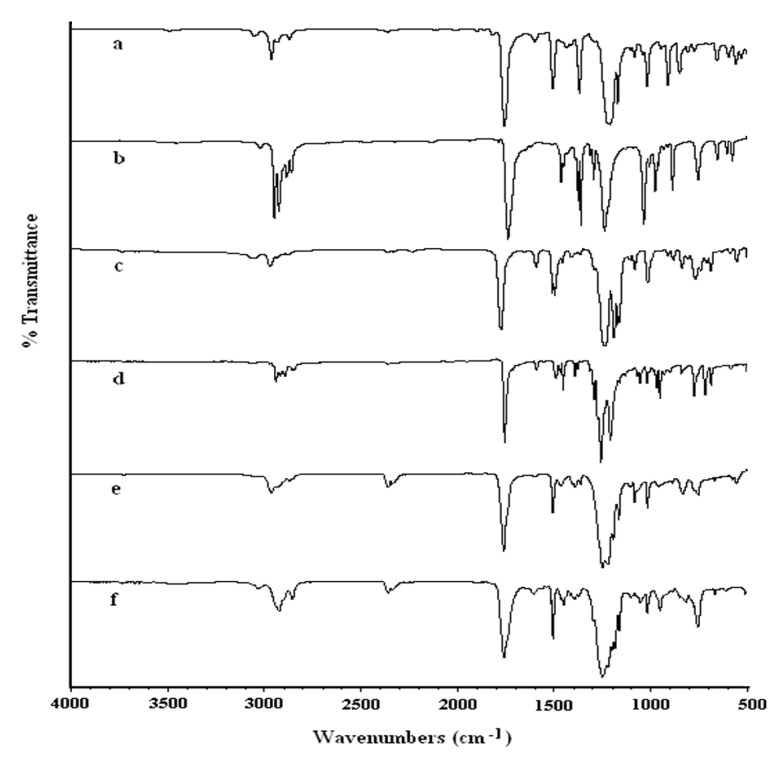
FTIR spectra of (a) BPA diacetate (b) CHDM diacetate (c) BPA DPDC (d) CHDM DPDC (e) BPA–PeD polycarbonate (f) CHDM-BHPM polycarbonate.

The FTIR spectra of BPA diacetate and CHDM diacetate (Figure 2 and Table 1) showed strong absorption bands due to the C=O stretching vibration of the ester group at 1,755 and at 1,741 cm^-1^, respectively.

The absorption bands of the C-O-C stretching vibrations of BPA diacetate were observed at 1,170 and 1,210 and that of CHDM diacetate at 1,239 cm^-1^. The IR spectra of diphenyl dicarbonate monomers (Figure 2 and Table 1) showed strong absorption bands due to the C=O stretching vibration of the carbonate group from 1,768 to 1,787 cm^-1^ for arylene diphenyl dicarbonates, and from 1,754 to 1,761 cm^-1^ for alkylene diphenyl dicarbonates. The absorption bands of the C-O-C stretching vibrations for both alkylene and arylene diphenyl dicarbonates were observed in the 1,158 to 1,271 cm^‑1^ range. These IR data are typical for the carbonate group [22,23,32,33,40] and thus suggest the formation of the proposed diacetate and diphenyl dicarbonate monomers in this work.

#### 2.1.2. NMR spectroscopy of monomers

*^1^H-NMR Spectra*: The ^1^H-NMR data for selected alkylene- and arylenediphenyl dicarbonates and diacetates are listed in Table 2. The ^1^H-NMR spectrum of BPA diacetate showed the aromatic protons of the BPA unit as an AB splitting system ranging from δ = 7.0 to 7.2. The methyl protons of the BPA unit were observed in the spectrum as a singlet at δ = 1.65, whereas the methyl protons of the acetate group were observed as a singlet at δ = 2.27. In the ^1^H-NMR spectrum of CHDM diacetate, the terminal methylene protons of attached to the ester group were observed as a doublet centered at δ = 3.81. The cyclohexylene ring tertiary CH protons were observed at δ = 1.74 whereas the CH_2_ ring protons were observed at δ = 1.0–1.7. The methyl protons of the acetate group were observed as a singlet at δ = 1.98. Regarding the diphenyl dicarbonate monomers, the ^1^H-NMR spectra of the following diphenyl dicarbonates: 1,2-ethanediol (EG DPDC), 1,3-propanediol (PrD DPDC), 1,4-butanediol (BuD DPDC), 1,5-pentanediol (PeD DPDC), resorcinol (RESOL DPDC), and hydro-quinone (HQ DPDC) were fully described in our previous publications [22,40]. The monomers: catechol diphenyl dicarbonates (CAT DPDC), and bis(4-hydroxyphenyl)methane diphenyl dicarbonates (BHPM DPDC), were introduced in this work. Here the spectra of the new diphenyl dicarbonate monomers utilized are only described. The ^1^H-NMR spectrum of CHDM DPDC showed a doublet peak for the protons of the terminal methylene group attached to the carbonate group at δ = 4.08.


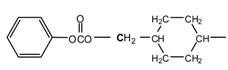


**Table 2:** ^1^H and ^13^C-NMR data for selected alkylene- and arylenediphenyldicarbonates and diacetates.

		Chemical shift δ (ppm)																				
		1		2		3	4	5		6		7		8		9		10		11		
**Dicarbonate**																						
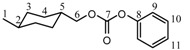	^1^H	4.08		1.91		1.0- 17.	1.0- 17.	1.91		4.08		—		—		a		a		a		
	^13^C	73.5		37.0		28.6	28.6	37.0		73.5		153.9		151.2		121.1		129.6		126		
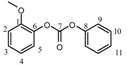	^1^H	-		a		a	a	a		—		—		—		a		a		a		
	^13^C	142.2		123		126.6	126.6	123		142.2		151.3		151		121		129.7		127.3		
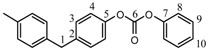	^1^H	4.0		—		a	a	—		—		—		a		a		a				
	^13^C	40.6		138.8		129.6	121	149.5		152.2		151		121		130		126.4				
**Diacetate**																						
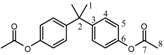	^1^H		1.65		—	—	7.20		6.97		—		—		2.27							
	^13^C		31.0		42.5	147.9	127.9		121		148.6		169.6		21.2							
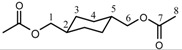	^1^H		3.81		1.74	1.0-1.7	1.0-1.7		1.74		3.81		-		1.98							
	^13^C		69.4		37	23.76	23.76		37		69.4		171.2		21							

^a^ The aromatic proton signals appear in the range δ = 7.1-7.5 as multiplets.

The peak for the tertiary CH ring proton was observed at δ = 1.91, whereas the CH_2_ ring protons were observed in the 1.0–1.7 range. The proton peaks of all arylenediphenyl dicarbonates were collectively observed as asymmetric multiplets interfering with the symmetrical signals of the various arylene units at δ = 7.1–7.5. In the spectrum of BHPM DPDC the aliphatic methylene protons of the BHPM unit (–C_6_H_5_–C**H**_2_–C_6_H_5_–) were observed as a singlet at δ = 4.0. The signals for the aromatic protons of the phenyl rings of the diphenyl dicarbonates were observed at δ = 7.1–7.4.

*^13^C-NMR Spectra*: The carbon signals of the BPA unit in BPA diacetate were observed in the spectra at the correct chemical shifts for this unit. The signal of the aliphatic quaternary carbon atom bearing the methyl groups appeared at δ = 42.5, while that of the methyl carbon atoms appeared at δ = 31.0. The signal of the aromatic quaternary carbon atoms of the rings next to the aliphatic quaternary carbon appeared at δ = 147.9. The signals of the quaternary carbon atoms of the aromatic rings attached to the ester group appeared at δ = 148.6. The signals of the carbon atoms of the ring in *ortho* and *meta* positions to the quaternary carbon atoms attached to the ester group appeared at δ = 121 and at 127.9. The signal of the methyl carbon atom of the acetate group appeared at δ = 21.2. The signal of the carbonyl carbon of the ester function was observed at δ = 169.6, which is the correct position for this type of carbonyl carbon.

In the ^13^C-NMR spectrum of CHDM diacetate, the peak of the terminal methylene carbon attached to the ester group appeared at δ = 69.4 ppm. The peak of the tertiary CH carbon atom of the cyclohexylene ring appeared at δ = 37.0. The peak of the methylene ring carbon atoms appeared at δ = 28.8. The signal of the methyl carbon atom of the acetate group appeared at δ = 21.0 ppm. The signal of the carbonyl carbon of the ester function appeared at δ = 171.2 ppm.

The ^13^C-NMR spectra of EG DPDC, PrD DPDC, BuD DPDC, PeD DPDC, RESOL DPDC, and HQ DPDC were fully described in our previous works [22,40]. The ^13^C-NMR spectrum of CHDM diphenyl dicarbonate showed peaks due to the terminal methylene carbon atoms (–OCOO**C**H_2_–) attached to the carbonate group at δ = 73.5. The peak of the tertiary CH carbon atom was observed at δ = 37.0 and that of the ring methylene carbon atoms at δ = 28.6, respectively. The aromatic catechol ring carbon atoms of catechol diphenyl dicarbonate attached to the carbonate group appeared at δ = 142.2 and those ortho to them at 123 whereas those meta to them at δ = 126.6. The peak of the aliphatic methylene carbon atom of BHPM diphenyl dicarbonate was observed at δ = 40.6. The peak of the quaternary aromatic carbon atoms attached to the aliphatic methylene carbon atoms was observed at δ = 138.8 while that of the quaternary aromatic carbon atoms attached to the carbonate group was observed at δ = 149.5. The peak of the carbonyl carbon of catechol and that of BHPM were observed at δ = 151.3 and 152.2, respectively.

### 2.2. Synthesis and characterization of polycarbonates

**Scheme 2:**
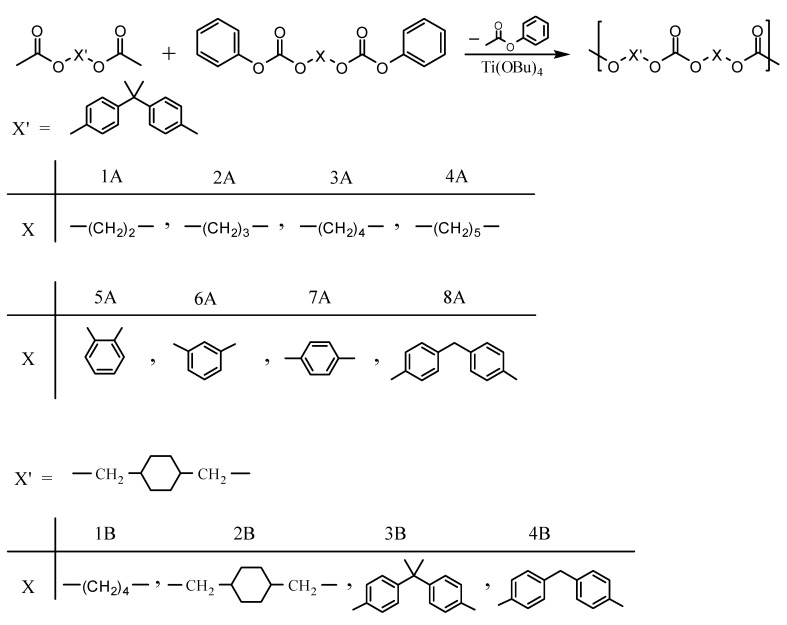
Synthesis of polycarbonate by melt polycondensation of alkylene and arylenediacetates with alkylene and arylenediphenyldicarbonates (Series A and B).

The various polycarbonates (Scheme 2) were synthesized from alkylene or arylene diacetates and alkylene- or arylenediphenyl dicarbonates. The polymerization reaction proceeded by the catalyzed nucleophilic reaction of the acetate group of the diacetate with the phenyl carbonate group of the diphenyl dicarbonate molecule using Ti(OBu)_4_ as the transesterification catalyst. The reaction afforded aromatic-aromatic, aromatic-aliphatic as well as aliphatic-aliphatic polymers. This assertion follows from the spectroscopic analysis of the formed polycarbonates especially the ^1^H-NMR and ^13^C-NMR spectra of the alkylene and arylene groups attached to the carbonate group and from the analysis of the carbonyl carbon peak of the carbonate group. It was found that conducting the polycondensation reaction at and above 240 °C did not afford any polymers, whereas at 220 °C it led to the formation of methanol insoluble polycarbonates.

#### 2.2.1. Solution viscosity measurements

The inherent viscosities of the polycarbonates were calculated from viscosity measurements of dilute polymer solutions (0.5 g/dL) in chloroform at 25 °C. The polycarbonates had inherent viscosities (Table 3) in the range from 0.19 to 0.43 dL/g. The synthesized polycarbonates had low to intermediate inherent viscosities, which implies that polymers with low to moderate molecular masses were obtained. The differences in the observed inherent viscosities (*i.e.*, molecular mass) may be attributed to the adverse effect of some factors.

An important factor that would limit the attainment of high molecular mass polycarbonates is the fact that the diphenyl dicarbonate and BPA diacetate compounds have unequal volatility at the polymerization conditions. This difference in volatility would slightly disturb the stoichiometric balance of the monomers in the reaction melt and would lead to polycarbonates with lower viscosities. The occurrence of other interchange reactions during polymerization is also another factor. The major and most reactive interchange reaction taking place for polymer chain buildup was the nucleophilic attack of the acetate group of BPA diacetate upon the phenyl carbonate group of the diphenyl dicarbonate monomer. This reaction yields linear polycarbonate chains in which the two monomer units are connected alternately along the entire length of the polymer chain. In addition to this reaction, another possible reaction was the self-condensation of the diphenyl dicarbonate monomer. Another possible competing reaction that could also occur was the partial elimination of cyclic aliphatic carbonates from the alkylene diphenyl dicarbonates or from already formed polymer chains. In fact, in this study, it was observed that EG DPDC or CAT DPDC reacting with bisphenol A diacetate, did not afford the postulated polycarbonate. In the synthesis of BPA–CAT polycarbonate, the formation of catechol cyclic carbonate was found to be the exclusive reaction occurring in the reaction melt. In addition to these reactions and under our experimental conditions, the formed polymer chains may undergo polymer-polymer, or polymer-monomer interchange reactions which would lead to viscosity alterations. In fact, these side reactions are essentially similar to the polycondensation reaction and would also occur under the same reaction conditions.

**Table 3:** Physical properties of polycarbonates as a function of the structure of the monomers.

Sample	Polycarbonate	Polym code	IR [v (cm^-1^)] C=O C-O		Inherent viscosity (η_inh_) (dL/g)	T_g_ (°C)
Series A						
1	BPA–EG^a^^)^	1A	—	—	—	—
2	BPA–PrD	2A	1,764	1,164, 1,194, 1,231	0.19	68.1
3	BPA–BuD	3A	1,761	1,164, 1,195, 1,245	0.23	56.5
4	BPA–PeD	4A	1,761	1,218, 1,250	0.24	49.5
5	BPA–CAT^ a)^	5A	—	—	—	—
6	BPA–RESOL	6A	1,777	1,164, 1,203	0.24	83.1
7	BPA–HQ	7A	1,772	1,163, 1,193, 1,228	0.26	97.0
8	BPA–BHPM	8A	1,772	1,161, 1,188, 1,231	0.27	107.8
Series B						
9	CHDM-BuD	1B	1,743	1,254	0.43	13.0
10	CHDM-CHDM	2B	1,743	1,252	0.36	38.7
11	CHDM-BPA	3B	1,761	1,216, 1,249	0.29	90.4
12	CHDM-BHPM	4B	1,760	1,185, 1,220, 1,251	0.24	56.9

^a)^ Polycarbonate soluble in methanol.

#### 2.2.2. Infrared spectroscopy

The FTIR spectra of the polycarbonates showed strong absorption bands due to the C=O stretching vibration of the carbonate group from 1,772 to 1,777 cm^-1^ for aromatic-aromatic polycarbonates and from 1,761 to 1,764 cm^-1^ for aromatic-aliphatic polycarbonates and at 1,743 cm^-1^ for aliphatic-aliphatic polycarbonates. Strong absorption bands due to the C–O–C stretching frequency for all polycarbonates were observed in the range from 1,161 to 1,254 cm^-1^ (Figure 2 and Table 3). These IR data are in accordance with the literature [22,23,32,33,42,43,44] and, therefore, confirm the formation of the various polycarbonates in this work. It was, however, observed that the C=O stretching vibration of aliphatic-aliphatic polycarbonate (1,743 cm^-1^) was very close to that of CHDM diacetate (1,741 cm^-1^). This confusion was resolved by the disappearance of the ^1^H-NMR signal of the methyl group and ^13^C NMR peak of the C=O of the ester group of the diacetate monomer in the NMR spectra of the final polycarbonates.

#### 2.2.3. NMR spectra of polycarbonates

*^1^H-NMR spectra*: The polymers synthesized in series A exhibit a similarity in the core structure, derived from BPA diacetate, but differ in the alkylene or the arylene parts of the various diphenyl dicarbonates. Therefore, the change in the pattern of the spectra could be correlated with the alkylene or arylene parts of the DPDC monomers in both aliphatic and aromatic polymers. The ^1^H-NMR spectra for the BPA unit were similar in terms of position and pattern of proton signals to those of the BPA diacetate for all polycarbonates. With regard to the aliphatic chains, the signal for the protons of the terminal methylene groups (–OOCOC**H**_2_–) attached to the carbonate group was observed in the range δ = 4.23–4.35 for all of the polycarbonates. The protons of the aliphatic methylene group next to the terminal methylene groups (–OOCOCH_2_C**H**_2_–) in the polycarbonates of BPA with Pr-1,3-D, Bu-1,3-D, and Pe-1,5-D were observed in the range δ = 1.77–2.14 with the correct pattern of splitting in each polycarbonate. In all ^1^H-NMR spectra of the polycarbonates, the peak of the protons of the methyl group of the acetate group, which was observed as singlet peak at δ = 2.27 in BPA diacetate was completely absent. The ^1^H-NMR spectra of the aromatic–aromatic polycarbonates were generally not indicative in confirming the chemical structures of these polycarbonates. The signals of the protons of the aromatic rings were collectively observed at δ values in the range δ = 7.1–7.3. The peak of the protons of the benzylic methylene group (–C_6_H_5_–C**H**_2_–C_6_H_5_–) of BHPM unit in BPA–BHPM polycarbonate was observed at δ = 3.92. These data, deduced from the ^1^H-NMR spectra, confirm the formation of aromatic aliphatic polycarbonates.

Parallel to series A polycarbonates, the chemical structure of the polycarbonates synthesized in series B consisted of a cyclohexanedimethylene unit bonded to different alkylene or arylene units through a carbonate group. The ^1^H-NMR spectra for the CHDM unit showed slightly shifted positions of proton signals of the CHDM diacetate monomer. The ^1^H-NMR spectrum of CHDM unit showed a doublet peak for the protons of the terminal methylene group attached to the carbonate group in the range from δ = 3.91–4.00. The peak for the tertiary CH ring proton was observed in the range from δ = 1.80–1.85 whereas the CH_2_ ring protons was observed in the same range at δ = 1.0–1.7. With regard to the aliphatic chains, the signals for the protons of the terminal methylene group (–OOCOC**H**_2_–) of BuD moieties attached to the carbonate group were observed at δ = 4.12. The protons of the aliphatic methylene group next to the terminal methylene groups (–OOCOCH_2_C**H**_2_–) was observed at δ = 1.73.


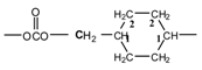


The proton signals of BPA and BHPM units in the polymers of series B were observed at approximately the same positions as those in series A. In all ^1^H-NMR spectra of the polycarbonates, the peak of the protons of the methyl of the acetate group, which was observed as singlet peak at δ = 1.98 in CHDM diacetate monomer, was completely absent. These data, deduced from the ^1^H-NMR spectra, confirm the formation of the various polycarbonates.

The disappearance of the proton singlet peaks of the methyl groups of the acetate function of both BPA diacetate (observed at δ = 2.27) and CHDM diacetate (observed at δ = 1.98) monomers from the ^1^H-NMR spectra of the various polycarbonates, indicates that the polycondensation reaction has occurred via these groups. Furthermore, the attachment of the terminal methylene group to the carbonate oxygen atom led to a downfield shift in the position of the signal in the spectrum to δ = 4.23–4.35. These facts were highly indicative and represent a spectral evidence of the formation of the various polycarbonates in this work.

*^13^C-NMR spectra*: The ^13^C-NMR spectra of the polycarbonates were quite informative and showed signals due to all aliphatic carbon atom types. Again the polymers synthesized exhibit a similar core structure derived from BPA diacetate, but differs in the alkylene or the arylene parts of the various diphenyl dicarbonates. The change in the pattern of the spectra could also be correlated with the alkylene or arylene parts in both aliphatic and aromatic polymers. The ^13^C-NMR spectra for the BPA unit were similar to those reported above for the BPA unit in BPA diacetate monomer for all polycarbonates.

With regard to the aliphatic chains of the polycarbonates in series A, the carbon atoms of the terminal methylene groups (–OOCO**C**H_2_–) attached to the carbonate group showed downfield effects similar to those observed in the ^1^H-NMR spectra and their signals appeared in the spectra of the polycarbonates of BPA with Pr-1,3-D, Bu-1,4-D, and Pe-1,5-D in the range δ = 65.1–68.5. The signals of the aliphatic methylene carbon atoms next to the terminal methylene carbons (–OOCOCH_2_**C**H_2_–) appeared in the range δ = 25.2–28.3.

The ^13^C-NMR spectra of the aromatic-aromatic polycarbonates, also confirmed the chemical structures of these polycarbonates. The signal of the quaternary carbon atoms of the aromatic rings of the dihydroxy compounds attached to the carbonate group appeared at δ = 148.3–151.4. The signal of the quaternary carbon atoms of the aromatic rings linked to the aliphatic quaternary carbon atom in BPA and in BHPM units, appeared in the range δ = 139–149. The signals of the carbon atoms of the aromatic rings of all aromatic dihydroxy compounds in ortho and meta positions to the quaternary carbon atom appeared in the range δ = 114–130. The peak of the carbon atom of the aliphatic methylene group (–C_6_H_5_–**C**H_2_–C_6_H_5_–) of BHPM unit in BPA–BHPM polycarbonate was observed at δ = 40.6. The characteristic signal of the carbonyl carbon of the carbonate group in all polycarbonates appeared in the range δ = 152.2–153.9. In all ^13^C-NMR spectra, the signals of the methyl carbon atom of the methyl acetate group and the carbonyl carbon of the ester group of BPA diacetate were completely absent.

Regarding the ^13^C-NMR spectra of series B polycarbonates, the ^13^C-NMR spectra for the CHDM unit showed slightly shifted positions for the carbons peaks of the CHDM diacetate monomer.


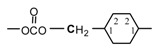


**Figure 3:**
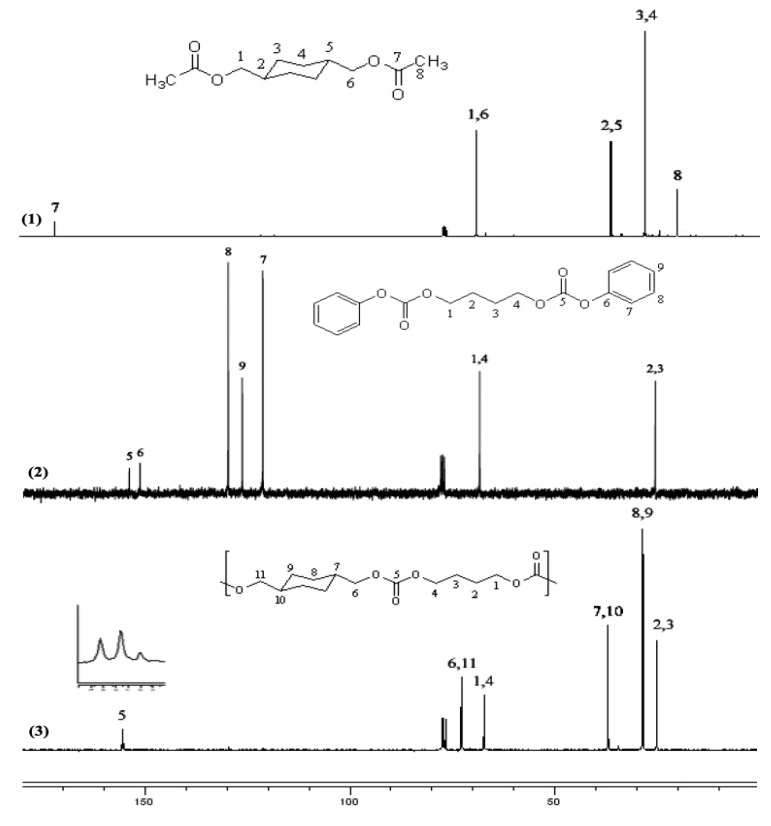
^13^C-NMR spectra of: (1) CHDMAc2 (2) BuD DPDC (3) CHDM-BuD polycarbonate (Series B).

The signal of the terminal methylene carbon atoms of cyclohexanedimethylene unit attached to the carbonate group appeared in the range δ = 72.8–73.1. The signal of the tertiary CH carbon atom and the methylene carbon atoms CH_2_ of the cyclohexylene ring, labeled 1 and 2 appeared at δ = 37.0 and δ = 28.6, respectively. With regard to CHDM-BuD polycarbonate, the terminal methylene carbon atoms of the BuD moiety (–OOCO**C**H_2_–) attached to the carbonate group and those next to them (–OOCOCH_2_**C**H_2_CH_2_–) showed their peaks at δ = 67.3 and δ = 25.2, respectively. The signals of the aromatic and aliphatic carbon atoms of BPA in CHDM-BPA polycarbonate and those of BHPM in CHDM-BHPM polycarbonate as well as their quaternary carbon atoms appeared approximately at the same positions as those observed in series A polycarbonates. The signal of the carbonyl carbon of the carbonate group in the polycarbonates of series B appeared in the range δ = 153.9–155.5. In all ^13^C-NMR spectra of the polycarbonates synthesized, the signal of the methyl carbon atom of the methyl acetate group of CHDM diacetate and the carbonyl carbon of the ester group, which appeared at δ = 21 and 171.2 ppm, were completely absent. The ^13^C-NMR spectra of CHDM diacetate, BuD DPDC and CHDM-BuD polycarbonate (Series B) are compared in Figure 3.

The disappearance of peaks 7 and 8 of CHDM diacetate (Figure 3, spectrum 1) and peaks 7, 8 and 9 of BuD diphenyl dicarbonate (Figure 3, spectrum 2) from the ^13^C-NMR spectrum of the CHDM-BuD polycarbonate (Figure 3, spectrum 3), indicates that the polycondensation reaction has occurred via the methyl acetate and the phenyl carbonate groups of the monomers. This fact also represents a spectral evidence of the formation of the various polycarbonates in this work.

#### 2.2.4. ^13^C-NMR spectral evidence of unit sequence randomization

**Figure 4:**
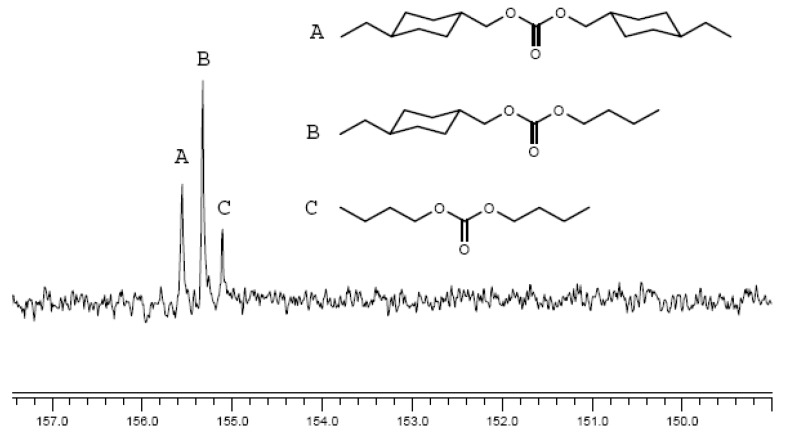
^13^C-NMR peaks of C=O observed in the spectrum of CHDM–BuD polycarbonate.

In addition to the above findings, a spectral evidence for the existence of structural variations within the polymer backbone of the polycarbonates was also observed. An interesting pattern of peaks observed in the ^13^C-NMR spectra of almost all polycarbonates synthesized was the appearance of three peaks for the carbon atom of the carbonate group (C=O) in the range δ = 151–156 ppm instead of a single peak for the perfectly alternating sequence of monomer residues. For example, the ^13^C-NMR spectrum of CHDM-BuD polycarbonate:

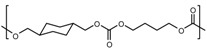

displayed three peaks for the carbonyl carbon (C=O) of the carbonate function, as illustrated in Figure 4. In order to interpret the peaks, poly(1,4-tetramethylene carbonate) homopolymer was synthesized from BuAc2 and Bu DPDC and its ^13^C-NMR spectrum was recorded. When the ^13^C-NMR spectra of poly(1,4-tetramethylene carbonate) homopolymer was compared to that of poly(CHDM carbonate) homopolymer (polycarbonate 2B in Scheme 2 series B), the C=O carbon peak positions of the carbonate groups were observed at 155.20 and at 155.52 ppm, respectively. The observed pattern was, therefore, accounted for as in the Figure 4.

This pattern proves that the backbone of the polycarbonates synthesized by interchange reactions possessed a partial random sequence of units in which all of these sequences coexisted. Peak B in the spectrum represents the alternate sequence of monomer residues in the polymer backbone. This peak should be due to the sequence resulting from the direct reaction of the diacetate with the diphenyl dicarbonate monomers. The occurrence of the random sequences A and C may have been due to other interchange reactions such as self-condensation of DPDC monomers, elimination of cyclic aliphatic carbonates from polymer chains, polymer-polymer and polymer-monomer reactions.

In order to expand the discussion of the subject matter of the present method, it may be advantageous to compare the results of the present method to those of interchange reactions of dihydroxy compounds with alkylene- and arylenediphenyl dicarbonates. The results of the diacetate-diphenyl dicarbonate reaction indicated that this reaction generally afforded lower reaction yields and lower polymer inherent viscosities (*i.e.*, lower molecular mass) than the diol-diphenyl carbonate reaction. The diacetate and the diphenyl dicarbonate monomers vary in their volatility so it is difficult to maintain a good stoichiometric balance of monomers throughout the reaction. Furthermore the reaction is accompanied by a yellow discoloration which is more intense than in the case of diol-diphenyl dicarbonate reaction. This discoloration becomes more intense with increasing the temperature and heating time. Finally, polycarbonates were not obtained from diacetates and diphenyl dicarbonates at temperatures in excess of 240 °C. This disadvantage precludes achieving high molecular mass polycarbonates.

### 2.3. Thermal properties

**Figure 5:**
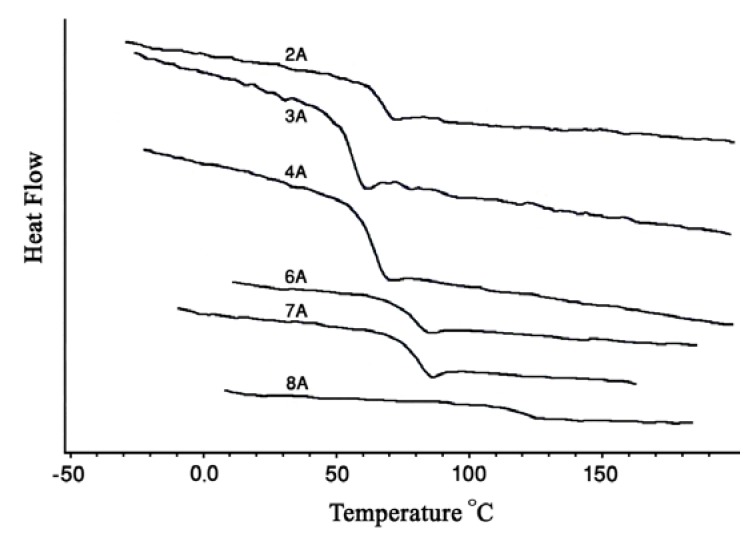
DSC Thermograms of polycarbonates (Series A).

**Figure 6:**
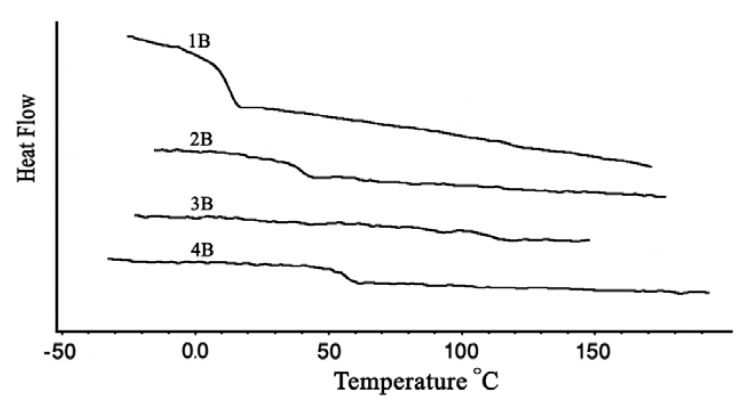
DSC Thermograms of polycarbonates (Series B).

The thermal properties of polycarbonates synthesized were investigated with DSC and TGA. The glass transition temperature (T_g_) values of the aromatic-aromatic polycarbonates were generally higher than those of aromatic-aliphatic polycarbonates (Figure 5 and Figure 6). The T_g_ values of the polycarbonates (Table 3) were in the range 13–107.8 °C. The aromatic-aromatic polycarbonates showed T_g_ values in the range 83.1–107.8 °C, aromatic-aliphatic polycarbonates in the range 49.5–90.4 °C, and aliphatic-aliphatic polycarbonates showed values at 13 and at 38.7 °C. The high T_g_ values of aromatic-aromatic polycarbonates may have been due to the stiffening effect of aromatic groups which restricted the movement of the polymer chains and thus increased T_g_. The presence of aliphatic segments in the polymer backbone imparted flexibility to move under the effect of temperature. This ease of motion was reflected in the lower T_g_ values of aromatic-aliphatic polycarbonates.

**Figure 7:**
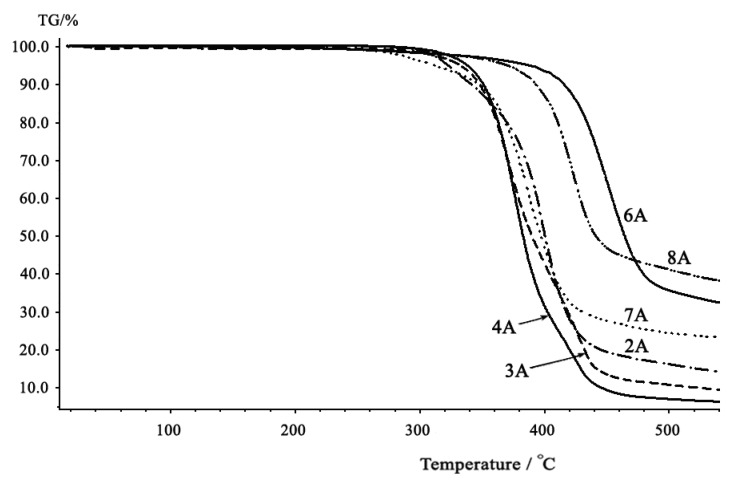
TGA thermograms of polycarbonates (Series A). TG % is the mass percentage of the polymer sample remaining after heating the polymer to a high temperature.

**Figure 8:**
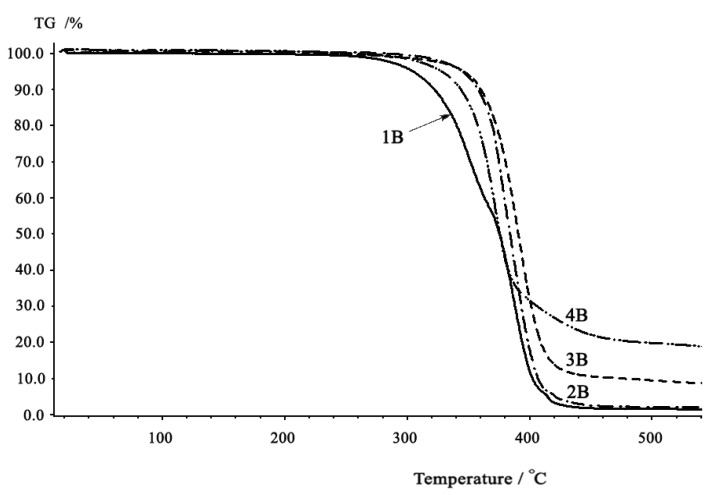
TGA thermograms of polycarbonates (Series B). TG % is the mass percentage of the polymer sample remaining after heating the polymer to a certain temperature.

As shown in the TGA thermograms (Figure 7 and Figure 8), the thermal decomposition temperatures (T_d_) of aromatic-aromatic polycarbonates were considerably higher than those of aromatic-aliphatic or aliphatic-aliphatic polymers. They also have higher residual mass percentages at 500 °C. The TGA curves of the polycarbonates displayed a slow mass loss starting between 250–400 °C and a relatively fast mass loss occurring between 350–500 °C. In particular, the thermogravimetric curves of aromatic-aromatic polycarbonates showed that these polymers have good thermal stabilities and displayed typical one-stage characteristics with a relatively fast mass loss occurring at temperatures higher than those of the aromatic-aliphatic or aliphatic-aliphatic polycarbonates.

## 3. Experimental

### 3.1. Materials

Reagents and chemicals are all commercially available and were used without any further purification. The following chemicals were purchased from the corresponding companies: ethylene glycol, 1,4-butanediol, hydroquinone, and phenyl chloroformate from Merck (Schuchardt, Germany); 1,3-propanediol, titanium(IV) *n*-butoxide, 4,4'-isopropylidenediphenol, 1,4-cyclohexanedimethanol, 4-dimethylaminopyridine, and 4,4'-isopropylidenediphenol diacetate from Acros Organics (New Jersey, USA); bis(4-hydroxyphenyl)methane from Aldrich (Milwaukee, WI, USA); resorcinol from Riedel-de Haën (Seelze, Germany); and 1,5-pentanediol from Lancaster (Morecambe, England). 1,4-Cyclohexanedimethylene diacetate was prepared and characterized in our laboratories as described in the experimental part. Chloroform was dried over diphosphorus pentoxide. Tetrahydrofuran was purified by refluxing and distillation over sodium wire and benzophenone. Zinc acetate dihydrate was dried at 100 °C under vacuum overnight.

### 3.2. Monomer synthesis

*1,4-Cyclohexanedimethylene diacetate:* The diacetate was prepared from 1,4-cyclohexane dimethanol and acetyl chloride according to the following procedure: acetyl chloride (7.85 g, 0.10 mol) in chloroform solution (30 mL) was added dropwise to a stirred solution of 1,4-cyclohexanedimethanol (7.21 g, 0.050 mol), pyridine (17.40 g, 0.22 mol) and a catalytic amount of 4-dimethylaminopyridine (4-DMAP) in chloroform (60 mL) below 5 °C. The reaction mixture was stirred for 1 h at 0–5 °C and overnight at room temperature, and then washed with 5% HCl solution and 2 × 100 mL of water. The chloroform layer was poured onto distilled water (600 mL) yielding the 1,4-cyclohexanedimethylene diacetate as solid crystals, which were collected by filtration, washed with 2 × 60 mL of distilled water and dried at 30°C under reduced pressure to yield white crystals (8.71 g, 87% yield, T_m _= 70°C).

*Alkylene and arylene diphenyl dicarbonates*: The diphenyl dicarbonate monomers were prepared by the reaction of the corresponding dihydroxy compounds with phenyl chloroformate as shown in Scheme 1, following previously published procedures [33]. A brief general synthetic procedure for the preparation of 1,5-pentanediol diphenyl dicarbonate (PeD DPDC) follows: 

Phenyl chloroformate (15.66 g, 0.10 mol) in dry THF (20 mL) was added dropwise to a stirred solution of 1,5-pentanediol (5.21 g, 0.050 mol), pyridine (9.50 g, 0.12 mol) and a catalytic amount of 4-dimethylaminopyridine (4-DMAP) in THF (350 mL) at 0–5 °C. The reaction mixture was stirred for 1 h at 0–5 °C and overnight at room temperature, and then poured into distilled water (600 mL). The precipitate formed was collected by filtration, washed several times with 10% aqueous sodium carbonate solution; the product was purified by recrystallization from ethyl acetate and dried at 50 °C under vacuum overnight to give white crystals (14.98 g, 87 % yield, T_m _= 52 °C).

The following diphenyl dicarbonates of the other dihydroxy compounds were prepared by the same procedure: ethylene glycol diphenyl dicarbonate (EG DPDC), 1,3-propanediol diphenyl dicarbonate (PrD DPDC), 1,4-butanediol diphenyl dicarbonate (BuD DPDC), 1,4-cyclohexanedimethanol diphenyl dicarbonate (CHDM DPDC), catechol diphenyl dicarbonate (CAT DPDC), resorcinol diphenyl dicarbonate (RESOL DPDC), hydroquinone diphenyl dicarbonate (HQ DPDC), bis(4-hydroxyphenyl)methane diphenyl dicarbonate (BHPM DPDC), and bisphenol A diphenyl dicarbonate (BPA DPDC). The yields and physical properties of these compounds are listed in Table 1.

### 3.3. Polycarbonate synthesis

The polycarbonates were synthesized from BPA diacetate and the various diphenyl dicarbonates (Scheme 2) according to the following general procedure: a glass reaction tube (150 × 30 mm outside diameter) equipped with a gas inlet and outlet and stop cocks, was filled with a homogeneous solid mixture composed of 10 mmol BPA diacetate, 10 mmol diphenyl dicarbonate and Ti(OBu)_4_ (2% mol of the dicarbonate). The reaction mixture was heated in a silicone oil bath at 160 °C under a slow stream of argon gas for 30 min. The temperature of the bath was then heated at 170 °C, at 190 °C and then at 200 °C for 1 h for each temperature. The reaction mixture was further heated to 220 °C and kept at this temperature for 1 h. While maintaining the temperature at 220 °C the gas inside the tube was gradually evacuated over a period of 1 h, and heating was maintained for 1 h. Finally, the reaction tube was cooled, the formed polymer was dissolved in chloroform, the solution was filtered and the polymer precipitated by drop wise addition to methanol. The polymer was filtered and dried in vacuo in a drying pistol at 60 °C over night.

The same procedure was followed for the preparation of the following polycarbonates: BPA–PrD polycarbonate, BPA–BuD polycarbonate, BPA–PeD polycarbonate, BPA–RESOL polycarbonate, BPA–HQ polycarbonate, and BPA–BHPM polycarbonate 

Two series of polycarbonates were prepared as demonstrated in Scheme 2:

1) Series A: was prepared from the reaction of BPA diacetate with alkylene and arylenediphenyldicarbonates.
2) Series B: was prepared from the reaction of 1,4-cyclohexanedimethylene diacetate with alkylene and arylenediphenyldicarbonates.

### 3.4. Monomer and polymer characterization

*Inherent viscosity measurements*: The inherent viscosity of polymer solutions in chloroform were measured with a dilution Ubbelohde glass capillary viscometer (Rheotek, Poulten Selfe & Lee Ltd., Essex, England) in a thermostated water bath temperature at 25 °C. The solutions were temperature equilibrated for approximately 10 min before viscosity was measured.

*Molecular characterization*: The Fourier transform infrared (FTIR) spectra (Spectral resolution: 4 cm^-1^) of the monomers and polymers were recorded as neat films with Thermo Nicolet Nexus 670 FT-IR spectrophotometer (Madison, WI, USA). The films were prepared by cast solution of purified monomer or polymer in chloroform over NaCl plates. The ^1^H-NMR and ^13^C-NMR spectra of the monomers and polymers were recorded with a Bruker Avance DPX 300 FT-NMR Spectrometer at 300 MHz (Wissembourg Cedex, France) in deuterated chloroform. Chemical shifts (δ) for ^1^H and of 75 MHz for ^13^C are given in ppm with respect to tetramethylsilane (TMS) as the standard.

### 3.5. Thermal properties

The glass transition temperature (T_g_) values of polymer samples were studied with a Netzsch DSC 204 F1 Differential Scanning Calorimeter (Selb Bavaria, Germany). The T_g_ measurements were conducted on 10 ± 2 mg samples under dry nitrogen. The samples were first heated to 150 °C and maintained for 2 minutes before rapid cooling with liquid nitrogen to ambient or sub-ambient start temperature. The thermal behaviors of the samples were probed by heating to the molten state at a heating rate of 20 °C/min for aromatic-aromatic polycarbonates and 10 °C/min for aromatic-aliphatic polycarbonates. The T_g_ values were taken as the mid point of the step transition. The thermal stabilities of the polymer samples were studied by thermogravimetric analysis (TGA) with a Netzsch STA 409 PG/PC thermal analyzer (Selb Bavaria, Germany). Measurements were conducted at a heating rate of 20 °C/min under dry nitrogen atmosphere purging at a flow rate of 50 mL/min.

## 4. Conclusions

In the present study, a new method of synthesis of polycarbonates, useful as engineering plastics, based on reaction of alkylene and arylene diacetate with alkylene- and arylenediphenyl dicarbonates in the molten state was presented. The inherent viscosity range obtained utilizing the present method was 0.19–0.43 dL/g, which implies that the polycarbonates with low to intermediate molecular mass were formed. The potential of this method was demonstrated by the successful synthesis of several polycarbonates prepared in this study. The ^13^C-NMR spectra of the carbon of the carbonate group showed that the formed polycarbonates contain a partial random sequence distribution of monomer residues in their chains. With this approach, it was possible to provide a flexible method of synthesis of polycarbonates whose properties could be varied by varying the structure of the diacetate or the dicarbonate monomers. Compared to polycarbonates obtained by diol-diphenyl dicarbonate interchange reaction, the method practically afforded polymers with lower yields and smaller molecular mass.
